# A Novel MAPT Mutation, G55R, in a Frontotemporal Dementia Patient Leads to Altered Tau Function

**DOI:** 10.1371/journal.pone.0076409

**Published:** 2013-09-27

**Authors:** Abhinaya Iyer, Nichole E. LaPointe, Krzysztof Zielke, Mariusz Berdynski, Elmer Guzman, Anna Barczak, Małgorzata Chodakowska-Żebrowska, Maria Barcikowska, Stuart Feinstein, Cezary Żekanowski

**Affiliations:** 1 Neuroscience Research Institute and Department of Molecular, Cellular and Developmental Biology, University of California Santa Barbara, Santa Barbara, California, United States of America; 2 Department of Psychiatry, 105 Military Hospital with Outpatient Clinic in Zary SPZOZ, Żary, Poland; 3 Laboratory of Neurogenetics, Mossakowski Medical Research Center, Polish Academy of Sciences, Warszawa, Poland; 4 Neurology Clinic, MSWiA Hospital, Warszawa, Poland; Brigham and Women's Hospital, Harvard Medical School, United States of America

## Abstract

Over two dozen mutations in the gene encoding the microtubule associated protein tau cause a variety of neurodegenerative dementias known as tauopathies, including frontotemporal dementia (FTD), PSP, CBD and Pick's disease. The vast majority of these mutations map to the C-terminal region of tau possessing microtubule assembly and microtubule dynamics regulatory activities as well as the ability to promote pathological tau aggregation. Here, we describe a novel and non-conservative tau mutation (G55R) mapping to an alternatively spliced exon encoding part of the N-terminal region of the protein in a patient with the behavioral variant of FTD. Although less well understood than the C-terminal region of tau, the N-terminal region can influence both MT mediated effects as well as tau aggregation. The mutation changes an uncharged glycine to a basic arginine in the midst of a highly conserved and very acidic region. In vitro, 4-repeat G55R tau nucleates microtubule assembly more effectively than wild-type 4-repeat tau; surprisingly, this effect is tau isoform specific and is not observed in a 3-repeat G55R tau versus 3-repeat wild-type tau comparison. In contrast, the G55R mutation has no effect upon the abilities of tau to regulate MT growing and shortening dynamics or to aggregate. Additionally, the mutation has no effect upon kinesin translocation in a microtubule gliding assay. Together, (i) we have identified a novel tau mutation mapping to a mutation deficient region of the protein in a bvFTD patient, and (ii) the G55R mutation affects the ability of tau to nucleate microtubule assembly in vitro in a 4-repeat tau isoform specific manner. This altered capability could markedly affect in vivo microtubule function and neuronal cell biology. We consider G55R to be a candidate mutation for bvFTD since additional criteria required to establish causality are not yet available for assessment.

## Introduction

Frontotemporal dementia (FTD) is a group of neurodegenerative disorders characterized by atrophy of the frontal and temporal lobes. It is the second most common cause of dementia in patients under 65 years of age, after Alzheimer's disease (AD; [1]). The age of onset of FTD is usually between 45 and 65 years, although 10% of patients have an onset beyond 70 years of age [2]. Familial forms of FTD occur in 30–50% of cases, presenting as three distinct phenotypes known as (i) behavioral variant FTD (bvFTD), (ii) semantic dementia (SD) and (iii) progressive nonfluent aphasia (PNFA) [3], [4], [5]. The clinical characteristics of these illnesses are diverse, including loss of social skills, apathy, disinhibition, repetitive and compulsive behaviors, progressive inability to represent the self and others, loss of word meaning and the inability to express oneself. Parkinsonian signs are common, but typically emerge at a later stage of the disease [6]. The behavioral variant form of FTD manifests itself typically by disinhibition, compulsive or perseverative behavior, and apathy with emotional bluntness. MRI scans generally reveal gray matter loss, particularly in the anterior and medial temporal lobes, with less involvement of the parietal lobes, and typically no involvement of the cingulate gyrus or precuneus [7].

Of the 30–50% of FTD patients with a family history, approximately 10% exhibit an autosomal dominant mode of inheritance [8]. Mutations in seven genes can cause dominantly inherited FTD*: MAPT, GRN, TARDBP, FUS, VCP, CHMP2B and C9ORF72* [9]. The most common mutations occur in *MAPT, PGRN and C9ORF72*. However, more than 50% of the familial patients with FTD are not accounted for by any of these known disease genes.

Two types of mutations in the gene encoding the microtubule associated protein tau (*MAPT*) have been linked to FTD [10], [11], [12]. The first type is missense mutations that alter the amino acid sequence of the encoded tau protein. In contrast, the second type of *MAPT* mutations alters the pattern of tau alternative RNA splicing. Although most of these mutations are intronic and map to the splice junctions on either side of exon 10 (encoding part of the MT binding region of the protein; see below for details) and therefore do not affect the tau amino acid sequence itself, some exonic mutations alter both amino acid sequence and the pattern of alternative splicing. Thus, the genetics indicate that alterations in either tau structure-function or the regulation of tau alternative splicing can cause FTD. Interestingly, all three forms of FTD can be caused by *MAPT* mutations [13].

In order to gain insights into possible molecular mechanisms of pathological tau action, one must first consider normal tau action as well as its structure-function relationship. Tau is essential for the development and maintenance of the nervous system [14], [15], [16]. Among its many roles is to regulate the growing and shortening dynamics of microtubules (MTs; [17], [18], [19], [20], [21]), cytoskeletal polymers composed of tubulin dimer subunits that serve many essential cellular functions. MTs are especially important in highly elongated neuronal axons, where they assemble into parallel bundles that contribute to the establishment and maintenance of axonal morphology [22] and also serve as tracks for axonal transport [23]. In addition, tau is an important mediator of signal transduction [24], [25], [26].

**Figure 1:**
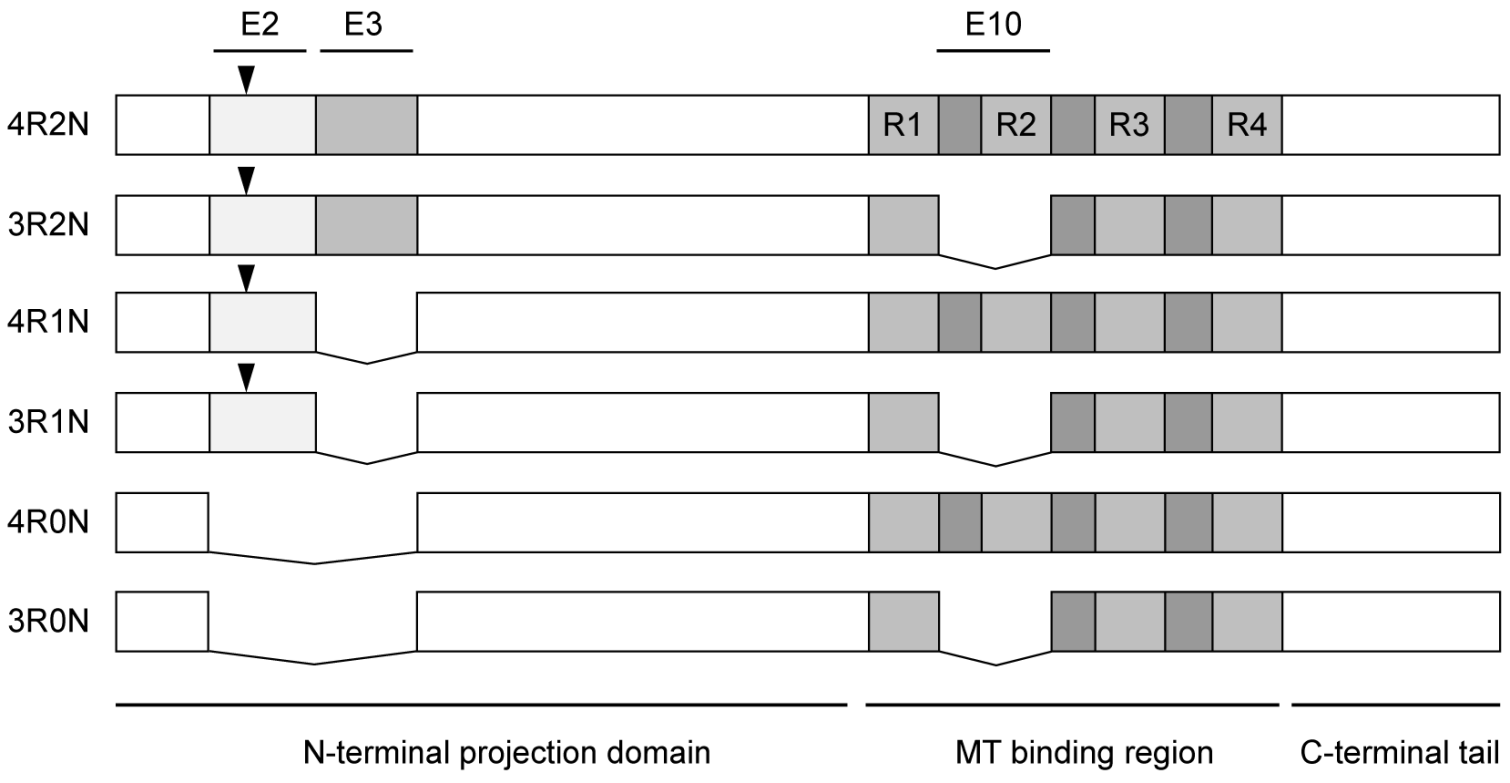
Schematic map of the six CNS tau isoforms. Exons 2 (E2), 3 (E3) and 10 (E10) are alternatively spliced to generate all six possible combinations. Arrowheads denote the position of the G55R mutation, present in four of the six isoforms. R1, R2, R3 and R4 denote the four imperfect repeats in the MT binding region.

Tau can be viewed as possessing three distinct structure-function regions (Figure 1). The best-characterized region is the MT binding region, which possesses either three or four imperfect repeats separated from one another by shorter inter-repeats [18], [27], [28], [29], [30], [31]. The distinction between 4-repeat (4R) tau and 3-repeat (3R) tau is determined by the inclusion or exclusion of exon 10 encoded sequences, determined by alternative tau RNA splicing [32]. In addition to MT binding capability, this region of tau also possesses inherent MT assembly promoting activity and the ability to regulate MT growing and shortening dynamics [17], [18], [19], [20], [21]. Generally speaking, 4R tau is a more potent mediator of these activities than is 3R tau [18], [30]. Importantly, this same region of tau has also been implicated in promoting pathological tau aggregation [33], [34], [35], [36].

On the N-terminal side of the MT binding region is a region known as the “projection domain” because it projects outward from the MT surface and determines the spacing between parallel, bundled MTs [37]. This region also serves (i) to indirectly influence the ability of the MT binding region to perform its microtubule-related functions [18], [29], [30], (ii) to self-aggregate in disease [38], [39], [40], (iii) to mediate the association of tau with membranes [41] and (iv) various roles in signaling, most notably as a substrate for kinases and phosphatases including the tyrosine kinase fyn [42], [43]. Further, this region has also been implicated in mediating tau dimerization [44], [45]. Of particular relevance to the work presented here, the projection domain contains zero, one or two inserts (29 amino acids each) encoded by exons 2 and 3 as a result of alternative tau RNA splicing (Figure 1; [46]). However, the functional differences between these three different tau isoforms (generally denoted as 0N, 1N or 2N, respectively) are poorly understood [47]. With respect to aggregation, 0N isoforms generally are less aggregation prone than longer isoforms [38], and exon 2 in particular appears to increase aggregation propensity [39]. Finally, the C-terminal tail of tau is located downstream of the MT binding region of tau. It is relatively short and is not well understood functionally. However, it can regulate tau binding to MTs indirectly [18], [30], likely at least in part via regulated phosphorylation [48].

Approximately 80% of the missense tau mutations causing FTD and/or related dementias such as PSP, CBD or Pick's disease map to the MT binding region, including many in the alternatively spliced exon 10 encoded sequences [49]. These mutations generally cause loss-of-function effects on the abilities of tau to bind MTs, promote MT assembly and regulate MT dynamics (for example, see [50], [51], [52]). Indeed, recent work demonstrating that MT stabilizing drugs such as taxol and epothilone D can reverse Aß mediated deficits in cultured neurons and disease symptoms in mouse models of Alzheimer's provide important support for this loss-of-function perspective [53], [54], [55]. However, many of these same mutations also increase the tendency of tau to form pathological oligomers and/or aggregates, consistent with a gain-of-toxic function perspective [33], [34], [35], [36]. It is important to note that these different perspectives need not be mutually exclusive.

There are five known tau missense mutations causing FTD or related dementias that map outside of the MT binding region of the protein. Two of these map to the extreme N-terminal end of the projection domain (R5H,R5L; [56], [57]) while the other three map to the C-terminal tail (K369I, G389R, R406W; [58], [59], [60]). The mechanistic effects of these mutations are poorly understood, especially the two mutations in the N-terminal projection domain. On the other hand, it has been speculated that the three mutations mapping to the C-terminal end of the protein might act indirectly by altering the ability of tau to serve as a substrate for regulatory kinases and/or phosphatases acting on amino acids mapping near to the mutations [51], [61]. Interestingly, no known tau mutations (prior to this work) map to the alternatively spliced exon 2 or exon 3 encoded sequences in the N-terminal region of the protein; this is in marked contrast to the many mutations mapping to the alternatively spliced exon 10 encoded sequences residing within the MT binding region of the protein.

Here, we describe the identification and characterization of a new tau mutation, G55R in a patient with bvFTD. G55R maps to exon 2, which encodes one of the two alternatively spliced inserts in the N-terminal region of the protein. In vitro analyses indicate that tau proteins bearing this substitution mutation promote more effective nucleation of new MTs when compared to WT tau, but only in the 4-repeat, adult specific tau context. This effect could significantly affect the regulation of MT action in adult neuronal cells. Thus, increased nucleation activity in 4R tau could underlie or contribute to pathological mechanisms associated with the G55R mutation. Identification of this novel candidate *MAPT* mutation, located in the N-terminal region of the tau protein, expands our understanding of tau molecular architecture and could contribute to the development of improved diagnostic and treatment strategies.

## Materials and Methods

### Ethics Statement

Written consent was obtained from the authorized proband's relatives and the diagnosed family members according to the Declaration of Helsinki (BMJ 1991; 302:1194). The genetic study was approved by the Ethics Committee of the CSK-MSWiA Hospital (Warszawa, Poland) in compliance with national legislation and the Code of Ethical Principles for Medical Research Involving Human Subjects of the World Medical Association. The same informed consent procedure was used for recruitment of the control groups.

### Genetic Analyses

Genomic DNA was extracted from peripheral blood leukocytes using standard procedures. Both strands of all *MAPT* amplicons were sequenced (exons 1–13 with flanking intronic sequences), as described previously [62]. Sequence data has been deposited in GenBank (accession number KC980907). The MAPT haplotype was determined by the presence or absence of a 238-base pair deletion between exons 9 and 10, as described by Baker et al. and then refined to sub-haplotypes using a panel of 5 SNPs (rs1467967, rs242557, rs3785883, rs2471738, rs7521; see [63], [64]).

### Generation and Purification of Tau and Kinesin

pRK expression vectors containing cDNA for the longest three-repeat and four-repeat human tau isoforms (3R2N tau and 4R2N tau; see Figure 1) were used in all studies. Quikchange site-directed mutagenesis (Stratagene, La Jolla, CA) was used to change glycine to arginine at position 55. All sequences were verified by DNA sequence analysis (Iowa State DNA Sequencing Facility). WT and mutant tau proteins were expressed in E.coli (DE3) and purified (>98%; see Figure S1 for representative images) as previously described [65]. Purified tau was dialyzed into BRB80 (80 mM PIPES, 1 mM EGTA, 1 mM MgSO_4_, pH 6.8, 0.1% ßME) and stored at −70°C. Concentrations of purified tau proteins were determined using densitometric comparison with a tau mass standard [19]. The kinesin construct K560-CL-his was a kind gift from Dr. Ron Vale (UCSF) and was purified as described [66] and then stored at −70°C in BRB80 supplemented with 10 mM βME and 0.1 mM ATP (Sigma, St. Louis, MO).

### Tubulin Purification and Labeling

Microtubule-associtated protein (MAP)-depleted tubulin was purified from bovine brain by two cycles of temperature-controlled polymerization and depolymerization, followed by phosphocellulose chromatography in PEM buffer (50 mM PIPES pH 6.8, 1 mM MgCl_2_, 1 mM EGTA) supplemented with 1 mM GTP (Sigma, St. Louis, MO) [67]. Aliquots of purified tubulin were drop frozen in liquid nitrogen and stored at −70°C. Protein concentrations were determined using the method of Bradford. SDS-PAGE analysis of the MAP-depleted tubulin stock revealed no detectable MAP contamination (data not shown). MAP-free bovine brain tubulin was labeled with 5-(and-6)-carboxyrhodamine 6G succinimidyl ester (Invitrogen, Eugene, OR) as previously described [68].

### MT Assembly Assays

MT assembly was assayed by light scattering in a modified 200 µL bulk phase turbidity assay [65]. Tau was added to cold BRB80 buffer (80 mM PIPES, 1 mM EGTA, 1 mM MgSO_4_, pH 6.8) containing 0.1% ßME to generate a final concentration of 0.75 µM. The diluted tau was mixed and transferred to wells in a 96-well plate on an ice block. MAP-free tubulin was thawed and added to a final concentration of 15 µM. GTP was added to a final concentration of 1 mM. Reactions were initiated by incubation at 37°C in a spectrophotometer and light scattering measured every 60 seconds at 340 nm for one hour. The first three data points were often unusable because of transient condensation on the plate.

### Co-sedimentation Assays

Assays were performed as described in Kiris et al. [65]. Tau-assembled MTs were harvested at the 60 minute time point of the MT assembly assays (see above). Samples were layered over 180 µL of a warm sucrose cushion (50% w/v in BRB80, 2 mM GTP) in a 5×20 mm UltraClear centrifuge tube (Beckman Coulter). Samples were centrifuged in a Beckman TLA 100.3 fixed angle rotor for 12 min at 60,000 rpm (153,000×g) at 35°C. Supernatants and pellets were collected and solubilized in SDS-PAGE sample buffer. The quantities of tau and tubulin in each fraction were determined by immunoblotting using the monoclonal antibody Tau-1 (Millipore) and Coomassie blue staining, respectively, taking care to operate within the linear detection range. Negligible quantities of tau or tubulin were present within the cushion. Statistical analysis of cosedimentation data was conducted using GraphPad Prism Software. Within each assembly condition, data were compared using a two-tailed t-test.

### Kinesin-Driven Microtubule Gliding Assays

MT gliding assays were performed as previously described [66]. A mix of fluorescent and unlabelled tubulin (15 µM) was assembled for 1 hr at 35°C in the presence of varying concentrations of WT or mutant tau (0.75 µM, 0.5 µM, 0.25 µM). Next, MTs were flowed into a kinesin-coated chamber slide, sealed with paraffin wax and imaged using a Nikon E800 fluorescent microscope and Metamorph software. Image analysis was performed using the Image J “Manual Tracking” plug-in (Fabrice P. Cordelieres, Institute Curie, Orsay, France). Velocities were calculated frame-by-frame and averaged for a single microtubule. ∼20 microtubules were analyzed per condition.

### MT Dynamics Assays

MT dynamics assays were performed as previously described [21], [52]. 15 µM MAP-free tubulin was assembled on the ends of sea urchin axonemes at 30°C in PMEM (96 mM Pipes, 45 mM MES, 1 mM EGTA, 2 mM MgCl_2_, pH = 6.8) containing 0.1% βME and 2 mM GTP for 30 minutes to achieve steady state. A 1∶60 tau∶tubulin ratio (0.31 µM tau) was used because previous cosedimentation data indicated that >90% of tau is bound to microtubules at this concentration (data not shown) and our previous work has demonstrated that this concentration of tau has significant effects upon the regulation of MT dynamics [21]. Time lapse images were obtained at 30°C by video differential interference contrast microscopy (DIC) using an inverted microscope with an oil immersion objective. Plus ends of microtubules were identified by their relatively fast growth rate and greater number of microtubules per axoneme relative to negative ends. MT dynamics of the plus ends were recorded for 40 minutes, capturing 10 minute videos per observation. Measurements of dynamicity were determined using RTM-II software and analyzed using Igor Pro software [69]. Growing events, shortening events, catastrophe, rescue, and overall dynamicity were measured using parameters previously described [70]. 20–30 microtubules were analyzed per condition. For each parameter, statistical significance was determined by comparing mutant tau to the corresponding WT using two-tailed t-tests in GraphPad Prism software.

### Aggregation Assays

Tau aggregation studies were carried out as described [33], [34], [35], [36]. For the 4-repeat isoforms, recombinant WT or G55R tau (2 µM) was diluted in aggregation buffer (100 mM NaCl, 10 mM Hepes, pH 7.4, 5 mM DTT, and 3.75% v/v ethanol), and aggregation was induced by the addition of arachidonic acid to 75 µM. Reaction mixtures were incubated for 24 h at room temperature and then samples were removed for electron microscopy. Samples were diluted 1∶10 into aggregation buffer containing 2% glutaraldehyde and fixed for 15 min. Fixed samples were adsorbed onto 300 mesh formvar carbon-coated electron microscopy grids for 1 min, and then negatively stained with 2% uranyl acetate.

Because 3-repeat tau constructs do not aggregate in vitro as readily as 4-repeat tau constructs, experimental conditions were modified to promote aggregation of 3R WT and 3R G55R tau. 3R WT and G55R mutant tau were used at 4 µM, aggregation was induced with 150 µM arachidonic acid, and reaction mixtures were incubated 5 h at 35°C. Samples removed for electron microscopy were added to 8% glutaraldehyde for a final concentration of 2% glutaraldehyde. Fixed samples were transferred to grids and stained as described above.

Grids were imaged at 20,000× magnification using a JEOL 1230 electron microscope operating at 80 kV, an ORCA digital camera and AMT Image Capture Software Version 5.24. Seven to ten fields were chosen from each grid under low magnification in order to prevent bias. Image J software (NIH) was used to threshold images and remove background noise. Finally, aggregates were measured and counted using the Analyze Particles function in Image J. Data from three independent experiments were analyzed by two-tailed, unpaired t-tests using GraphPad Prism statistical software.

### MT Length Distribution Assays

15 µm MAP-free tubulin was assembled in PMEM (96 mM Pipes, 45 mM MES, 1 mM EGTA, 2 mM MgCl_2_) with 0.1% βME and 2 mM GTP at 30°C for 1 hour, until steady state was reached. A 1∶60 tau∶tubulin ratio (0.25 µM tau) of either WT or mutant tau was used to assemble microtubules. After one hour, reactions were fixed in a 0.2% glutaraldehyde solution and placed on electron microscopy grids, stained with cytochrome c (1 mg/ml) and 1.5% uranyl acetate. Microtubules were imaged by electron microscopy at 3000× magnification, and a Zeiss MOP III morphometric digitizing pad was used to measure MT lengths. A minimum of 100 microtubules were measured for each experiment for a final total of at least 300 MTs per condition. Mean lengths for each condition were calculated, and statistical significance was determined using two-tailed t-tests to compare each mutant to the corresponding WT.

## Results

We describe a patient with a clinical diagnosis of frontotemporal dementia (behavioral variant) without Parkinsonsim and a novel heterozygous missense mutation (GGA>AGA, c.163g>a, BN000503.1) causing a substitution of arginine for glycine at position 55 (G55R) in the alternatively spliced exon 2 of the *MAPT* gene (Figure 1). The G55R mutation was absent in a group of 150 neurologically healthy subjects, aged >65 years, as well as in a group of 152 patients with clinically diagnosed familial and sporadic FTD. In the FTD group, two patients with the previously observed P301L mutation and one with the previously observed S305N mutation in the *MAPT* gene were also identified. In the G55R patient, we detected no mutation in the APP, PSEN1, or PGRN genes and no expansion in the C9ORF72 gene.

**Figure 2:**
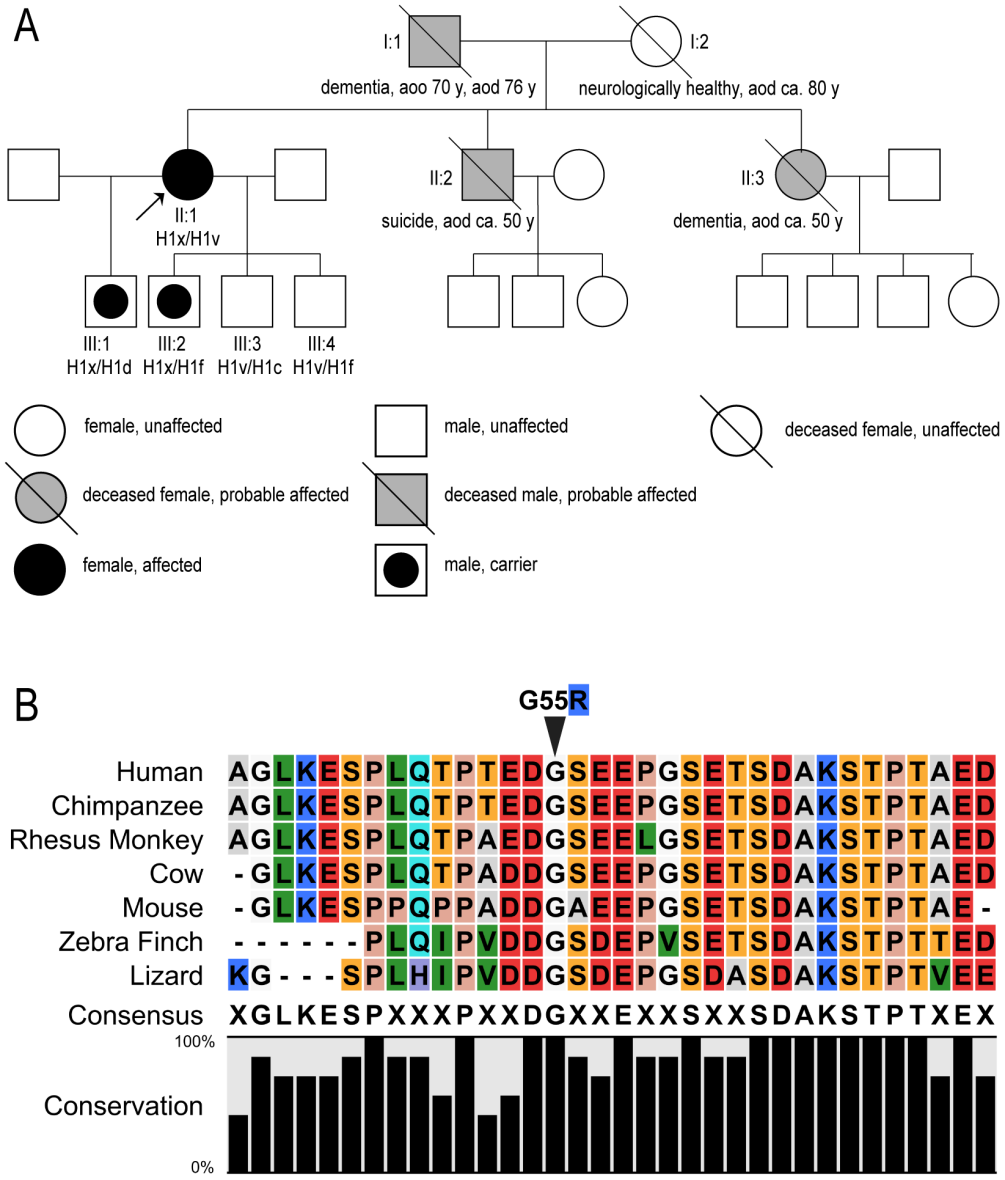
A. The family tree of the affected family shows the pattern of inheritance. The proband is the black oval on the left side of the figure (II:1), marked with an arrow. Tau haplotypes of sequenced individuals are also noted. “aoo” corresponds to age of onset; “aod” corresponds to age of death; black filling indicates persons possessing the G55R mutation; gray filling corresponds to diagnosed dementia of unknown origin (presumed to be G55R but inadequate medical records exist). Proband's son III:1 (from first marriage) is 36 years old and a carrier of G55R. Proband's second son III:2 (from second marriage) is 31 and also a G55R carrier. The other two sons (III:3 and III:4; from the second marriage) are not G55R carriers and are 29 and 28 years old. **B. The tau sequence in the region of the G55R mutation is extremely highly conserved across species lines.** The glycine at position 55 is completely conserved in seven species ranging from humans to lizards. Color coding emphasizes conserved nature of acidic (red), basic (blue), hydrophilic/polar (orange), hydrophobic (green) and proline (peach) positions.

Although the genetic evidence is incomplete, the family tree (Figure 2A) suggests a direct transmission of the disease from the proband's father, who exhibited similar symptoms of dementia and died in a nursing home from “general atherosclerosis” at the age of 76 years. The proband's sister (II:3) was a resident of a facility for people with mental disorders and required constant care. She died at the age of 50, most likely after years of dementia. The patient's brother (II:2) committed suicide at the age of 50 years and may or may not have also been affected. The proband has four children, two of whom carry the mutation (III:1, III:2), but they are not yet old enough to exhibit clinical symptoms. DNA sequencing demonstrated that the G55R mutation was in a haplotype H1x background in both the proband and her two sons.

### I. Clinical Case Report

A 55 year old, right-handed woman with elementary school education (8 years) reported three years of progressive behavior problems. Her medical history included ovarian cancer at age 49, resulting in removal of her uterus and its appendages. She was severely nicotine dependent. Her family reported that she had previously been sociable, jovial and enthusiastic. Subsequently, she became secretive, highly sensitive and suspicious along with the loss of the ability to perform routine functions. She also exhibited reduced appetite and became underweight (BMI 17). Gradually, she lost interest in daily activities, including watching television, meeting friends and walking, isolating herself from her environment. A problem with cutlery use occurred and gradually severe apraxia developed. She lost the ability to verbally express herself correctly and presented severe memory impairment. Anxiety, helplessness and sleep disturbances were also reported.

The patient was admitted to the psychiatric unit at a general hospital in December 2010. During this hospitalization, the patient exhibited anxiety and general disorientation. Limb and optical apraxia were observed. The patient experienced severe difficulty with word selection and speech (alogia, dysnomia, paraphrasia, and agrammatism). She expressed herself unclearly and indirectly, presenting a lack of insight and periodic verbal aggression. Psychotic symptoms were not observed. The patient spent most of her time pacing aimlessly through the ward. She also exhibited sleep and appetite disturbances. Clinical assessment revealed a MMSE score of 10 and a GDS score (Geriatric Depression Scale) of 8. At this point, she was diagnosed with frontotemporal dementia (FTD).

Seven months later, during a second hospitalization in the Neurology Clinic of the CSK MSWiA Hospital, the patient's condition was more advanced. She was withdrawn, exhibited reduced mobility, spent most of her time in bed (sitting or lying) and left her room only to smoke or use the rest room facilities (accompanied by her son). Her verbal expression was limited to partial, non-relevant answers to questions. She was very anxious and agitated when left alone. No verbal or physical aggression or psychotic disturbances were observed. A neurological examination revealed no abnormalities. On the other hand, neuroimaging analyses revealed generalized atrophy, located mostly in the frontal, parietal and temporal lobes, without ventricular enlargement and focal pathological changes. Neuropsychological testing was not possible because the patient's cognitive losses were too advanced (MMSE = 7). Co-operation during testing was impaired, caused by anxiety and agitation. She did not respond to questions, did not follow instructions and showed no involvement or interest in the requested tasks.

Ten months later, during a third clinical evaluation, the patient's condition had clearly deteriorated further. However, she was able to move on her own, albeit unsteadily. Her general mood was elevated. The patient had a tendency to shorten the distance between her and other people e.g. she tried to pinch medical personnel during examinations. Echolalia and confusion were observed. She was completely disoriented regarding place and time, and knew only her first and last names. She did not recognize her close relatives, claiming they were her friends. A clear helplessness was apparent. According to her daughters-in-law, she was not taking care of her personal hygiene, and did not want to bathe or to be washed. She did not use everyday items and was not able to dress herself. She often moved personal possessions from one shelf to another or hid them in strange places. From time to time, she showed verbal aggression. She occasionally had groundless, unexpected episodes of laughing. Her appetite was good but she was still losing weight (BMI 18). Her grade on a Global Deterioration Scale (GDS) was 6/7.

Based on the above observations, the patient was diagnosed with early-onset, familial frontotemporal dementia (behavioral variant) without parkinsonism.

### II. Molecular Analyses of G55R Tau

In the absence of more definitive genetic evidence, the G55R mutation must be designated at the present time as a candidate FTD mutation. However, bioinformatics approaches support the notion that the G55R mutation may cause tau dysfunction and therefore underlie the FTD symptoms. The G55R mutation changes an uncharged glycine residue into a basic arginine residue in the midst of a highly acidic region of the protein (Figure 2B). Additionally, analysis using the ConSurf (http://consurf.tau.ac.il), SIFT (http://sift.bii.a-star.edu.sg) and BLAST programs demonstrated that the G55 residue is highly conserved in evolution (Figure 2B) and that it is likely to be exposed on the surface of the protein, consistent with the unfolded nature of tau [71]. According to criteria proposed by Cotton and Scriver [72] and Antonarakis and Cooper [73], it is highly probable that G55R is a pathogenic variant of tau. The G55R mutation would also be classified as “probable pathogenic” according to the Guerreiro algorithm (used to assess AD-causing mutations [74]). Taken together, it is very possible that this G/R substitution could have marked structure-function effects, perhaps altering intra-molecular and/or inter-molecular interactions.

#### The G55R Mutation Increases the Ability of Tau to Nucleate Microtubule Assembly in 4R Tau but not 3R Tau

Tau action on MTs is the best characterized aspect of tau biochemistry. Therefore, our first efforts to characterize the effects of the G55R mutation were to compare the abilities of G55R tau and WT tau to promote MT assembly in vitro using a standard light scattering assay. We utilized “unseeded” reactions, which require tau to first nucleate new MTs and to then elongate them. The slope of the initial rise in light scattering is an indication of the efficiency of MT nucleation while the plateau level is a first approximation of the overall quantity of MTs assembled at steady state.

**Figure 3:**
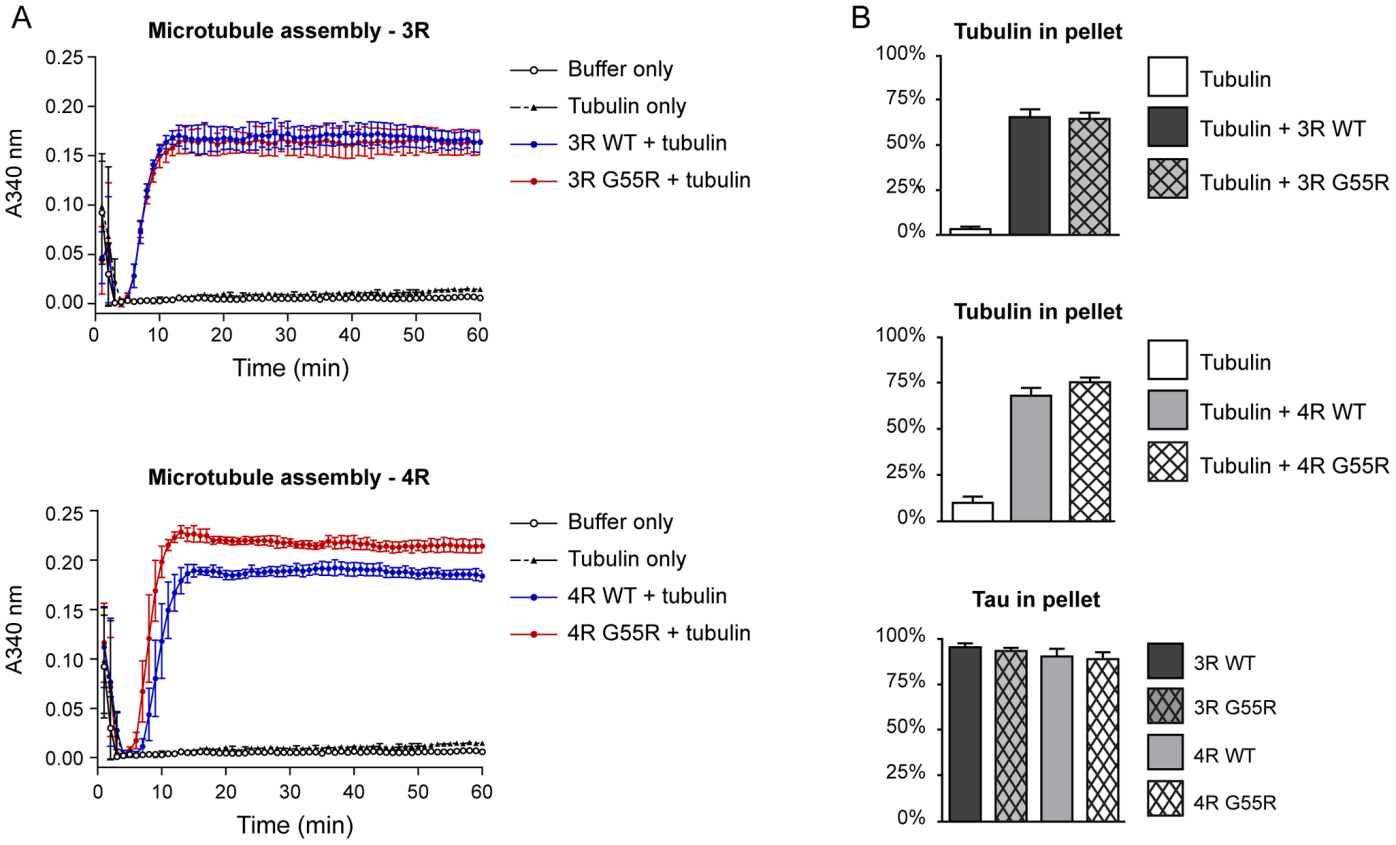
The G55R mutation increases the ability of 4R tau but not 3R tau to nucleate microtubule assembly. (A) Microtubule assembly in reactions containing a 1∶30 tau:tubulin dimer molar ratio were assayed by light scattering as a function of time. (B) Co-sedimentation assays demonstrate that the G55R mutation does not affect the ability of tau to assemble MT mass at steady-state, nor does it affect the ability of tau to bind to microtubules. Statistical significance was determined by comparing each mutant to its corresponding WT using two-tailed t-tests. Data in both panels represent the mean ± SEM from three independent experiments.

As seen in Figure 3 (panel A), the light scattering plots indicate that 4R G55R mutant tau nucleates and assembles MTs more effectively than does 4R WT tau. Quantitatively, the slope of the initial rise for the 4R G55R mutant tau plot (0.069±0.003 abs/min) is 32% steeper than the 4R WT tau control plot (0.047±0.006 abs/min; P = 0.035) and the plateau level for the 4R G55R mutant tau is ∼14% higher than for 4R WT tau (P = 0.0244). In contrast, the light scattering plots for 3R G55R tau and 3R WT tau are indistinguishable. Although perhaps surprising at first glance, there are other examples of tau mutations exhibiting differential effects in 4R tau versus 3R tau contexts [75].

Since the plateau level achieved in a light scattering MT assembly assay can be affected by both the number of MTs in a sample as well as the MT length distribution, we sought to independently assess the amount of MT mass assembled in each reaction shown in Figure 3A. Therefore, once steady-state had been achieved in the MT assembly assays (60 mins), aliquots were harvested from each reaction and subjected to co-sedimentation assays. Each sample was centrifuged through a sucrose cushion to pellet the MTs (and any bound tau) while leaving non-polymerized tubulin and non-MT bound tau in the supernatant. The amount of tubulin in each fraction was then determined by quantitative SDS-PAGE. As can be seen in Figure 3B, both 3R G55R tau and 4R G55R tau polymerize the same amount of MT mass as their corresponding WT tau proteins. Although the 4R G55R tau sample has a slightly greater amount of MT mass than 4R WT tau, this slight increase is not statistically significant. Additionally, the vast majority of tau in each case is MT bound. Taken together with the increased nucleation capability of 4R G55R demonstrated in Figure 3A, the simplest interpretation of the data is that 4R G55R tau nucleates a greater number of MTs than 4R WT tau but that the resulting MTs are shorter than those synthesized by 4R WT tau. This leads to the accumulation of the same overall MT mass and is the logical outcome of increased nucleation activity in an in vitro reaction with a finite quantity of tubulin subunits. In contrast, mutant 3R G55R tau and 3R WT tau exhibit the same levels of MT nucleation and assembly activities.

**Figure 4:**
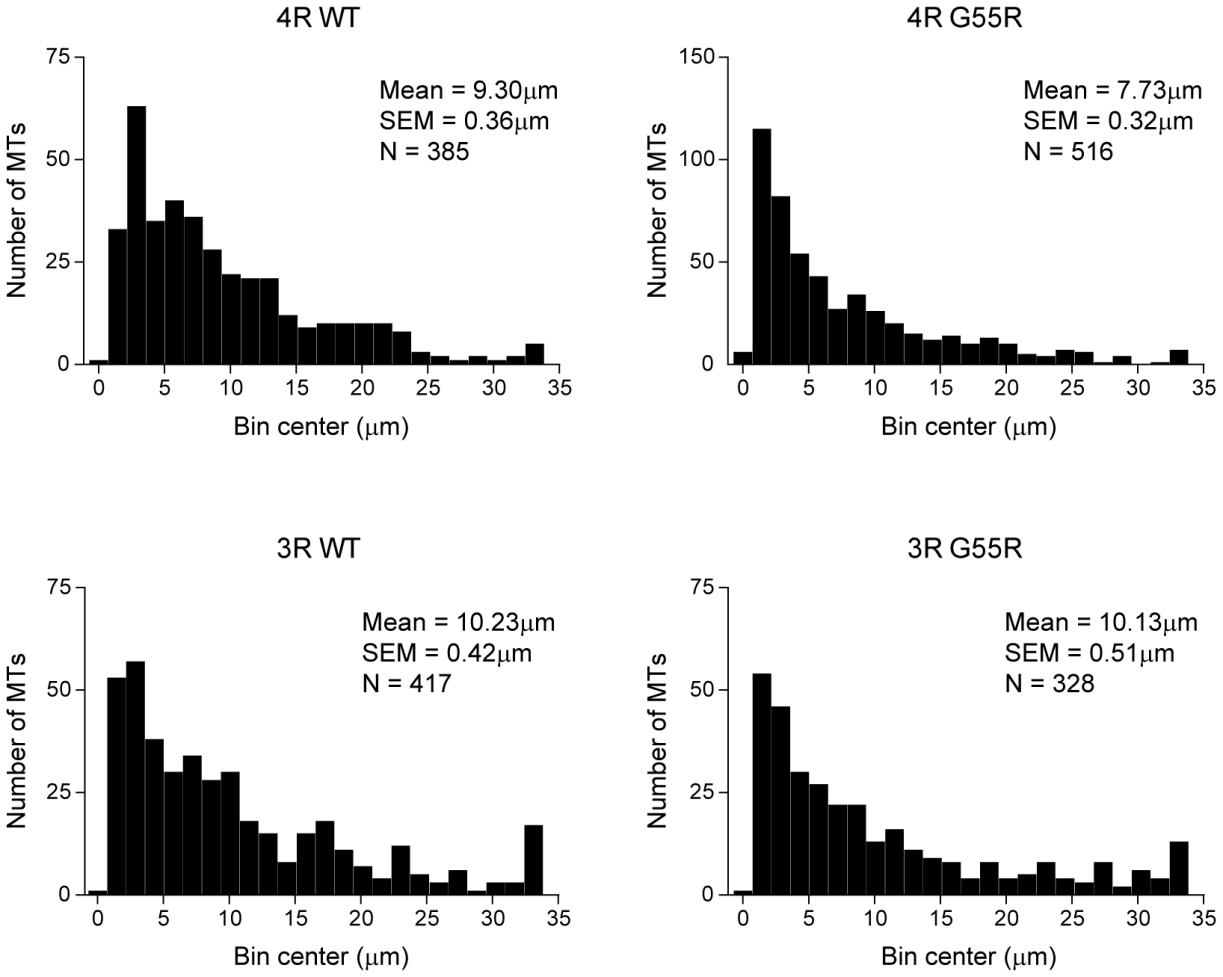
Length distribution of assembled microtubules. Aliquots of the microtubule assembly reactions shown in Figure 3 were harvested at the 60 minute time point and microtuble lengths were determined by transmission electron microscopy. Plots show binned MT lengths determined over three independent experiments, ± SEM. The mean length was calculated for each condition, and t-tests were used to compare each mutant to its corresponding WT.

To independently assess these conclusions, we next determined the length distribution of the MTs in each reaction at steady state using transmission electron microscopy (TEM). As seen in Figure 4, the mean length of MTs assembled by 4R G55R is 7.73 µm while 4R WT tau assembled MTs have a mean length of 9.30 µm, a statistically significant 17% difference (P = 0.001). In contrast, the mean length of 3R G55R tau MTs (10.13 µm) was statistically indistinguishable from 3R WT tau MTs (mean length  = 10.23 µm). Taken together with the data in Figure 3, these data corroborate the conclusion that the G55R mutation leads to increased MT nucleation activity in the 4R tau context, leading to the assembly of a greater number of MTs than 4R WT tau, although they have a shorter average length. In contrast, G55R has no effect on MT nucleation or average MT length in the 3R tau context.

#### The G55R Tau Mutation Does Not Affect the Ability of Either 4R Tau or 3R Tau to Regulate MT Dynamics or the Kinesin-MT Interaction

We next asked if the G55R mutation affected the ability of tau to regulate MT dynamics at steady-state using a standard in vitro MT dynamics assay system. We compared 4R G55R tau to 4R WT tau and 3R G55R tau to 3R WT tau for their abilities to affect MT growth and shortening rates, lengths of growing and shortening events, the durations of growth, shortening and attenuation (pause) events, the frequency of catastrophe and rescue events and overall dynamicity. Although there were some minor effects (Table S1), these did not reach statistical significance. The overall conclusion is that the G55R mutation does not have a marked effect upon the ability of tau to regulate MT dynamics. This conclusion is consistent with the MT assembly data derived above, since these MT dynamics assays use axoneme seeds to nucleate MT growth and therefore assess growing and shortening behaviors but not nucleation.

We next asked if the G55R mutation might affect the ability of tau-assembled MTs to interact with kinesin, the major axonal transport motor protein carrying cargo from neuronal cell bodies down the length of axons. We used standard kinesin-driven MT gliding assays to assess MTs assembled at tau:tubulin molar ratios of 1∶20, 1∶30 and 1∶60, and as seen in Figure S2, the gliding rates showed that G55R tau and WT tau assembled MTs behave indistinguishably from one another, in both 4R tau and 3R tau contexts.

#### The G55R Tau Mutation Does Not Affect The Ability of Either 4R Tau or 3R Tau to Aggregate

Since tau aggregation is a central feature of tau pathology, we next asked if the G55R mutation affected the ability of tau to aggregate using a standard aggregation assay. Tau aggregation was induced by the addition of arachidonic acid. Following incubation, the samples were fixed and examined by transmission electron microscopy. Aggregate number, aggregate size and total aggregate area were quantitated. Although there were occasional trends, there were no statistically significant differences between 4R G55R tau and 4R WT tau or between 3R G55R tau and 3R WT tau. Representative TEM images and data for total aggregate area per field are shown in Figure S3. (Data for aggregate number and aggregate size are not shown.) Although it is possible that a different inducer (such as heparin) might yield different results, or that a kinetic analysis might reveal differences in the rate of aggregation, these data suggest that the G55R mutation does not alter the ability of tau to aggregate.

These observations are consistent with our AGGRESCAN (http://bioinf.uab.es/aggrescan/) software analyses that identify putatively aggregation-prone regions in proteins. Our analyses revealed that the G55R mutation resulted in a negligible shift of a3v SA value (−0.331 vs. −0.332), which is an indicator of the amino acid aggregation-propensity value of the entire region. Additionally, position 55 was not identified as a hot spot for aggregation.

## Discussion

We have described a patient with the behavioral variant of FTD and a novel mutation (G55R) located in the alternatively spliced exon 2 of the *MAPT* gene, in the H1x haplotype background. This is the first tau mutation mapped to either of the two amino end tau exons (exons 2 and 3).

At this point in time, we consider G55R as a candidate gene for FTD because we cannot yet rigorously assess all requisite criteria required to establish causality. This follows from the facts that (i) DNA for genetic analyses of the proband's father and two siblings (all deceased) is not available, (ii) the proband is still alive and no brain tissue samples are available for immunocytochemistry and (iii) the proband's two sons carrying the G55R mutation are not yet of sufficient age to begin exhibiting symptoms. On the other hand, we believe that considerable evidence does exist to support the assertion that G55R tau is a very strong candidate to be causal for bvFTD. This includes the facts that (i) the dementia phenotype is inherited in a manner consistent with a simple dominant pattern of inheritance, consistent with other tau mutations causing FTD, (ii) the G55R mutation occurs at a very highly conserved position in tau, and the adjoining regions of tau are also very highly conserved, (iii) the glycine to arginine substitution changes an uncharged amino acid into a positively charged amino acid, right in the midst of a very negatively charged region of the protein, and (iv) the G55R mutation alters the ability of tau to nucleate MT assembly, one of the most important functions of the protein. Indeed, according to the Guerreiro algorithm (used to assess AD-causing mutations [74]), the G55R mutation would be classified as “probable pathogenic”. Using criteria proposed by Cotton and Scriver [72] and Antonarakis and Cooper [73], G55R would be highly probable to be a pathogenic variant.

### A New Candidate Tau Mutation for Frontotemporal Dementia

Despite the fact that the proband was diagnosed in an advanced stage of the disease, she met the stringent clinical criteria for probable bvFTD. She showed progressive deterioration of behavior and cognition and frontal and temporal atrophy by neuroimaging (MRI). The clinical phenotype of the G55R proband is significantly different from the phenotypes connected with the nearby R5L and R5H mutations that cause progressive supranuclear palsy and typical FTD, respectively [56], [57]. Additionally, the proband did not demonstrate any motor symptoms typical for CBD such as alien limb or myoclonus.

While the vast majority of tau mutations causing dementia map to the MT binding region of tau (Alzforum website; http://www.alzforum.org/res/com/mut/tau/table1.asp), G55R maps to the relatively poorly understood amino terminal region of the protein. Indeed, G55R is the first tau mutation mapping to either of the two alternatively spliced amino end tau exons (exons 2 and 3). More specifically, only two of the approximately two dozen previously characterized tau mutations are located in the amino half of the protein [56], [57]. Both of these map to amino acid position 5 (R5L and R5H). Both also exhibit a relatively late age of onset (75 and 62 years, respectively). In contrast, the proband and her two siblings (who we speculate carried the G55R mutation) presented with a relatively young age of onset (ca. 50 years). However the age of onset in the proband's father (who we also speculate carried the G55R mutation) was ca. 70 years. This may suggest the existence of additional factors modulating G55R phenotypic impact. One could speculate that the uncommon H1x haplotype associated with the G55R mutation could confer additional variability upon G55R action.

### Molecular Mechanisms of Pathogenesis

The molecular mechanisms underlying pathological tau action remain poorly understood and are a very active area of investigation. One key feature of this effort is the recognition that cells go to enormous lengths to tightly regulate tau activity. For example, relatively subtle errors in tau RNA alternative splicing having no effect on the tau amino acid sequence can cause neuronal cell death and dementia in FTD, PSP, CBD and Pick's disease [10], [11], [12], [76]. Subtle errors in the regulation of tau phosphorylation are also widely believed to play an important role in tau mediated neuronal cell death and dementia [48], [77]. By analogy to the pharmacology of MT directed anti-cancer drugs, we have proposed previously that cells must maintain tau activity within a narrow range of acceptable levels in order to remain viable [20], [21], [78]. By this mis-regulation of tau activity model, either too much tau activity or too little tau activity (or qualitatively altered tau activity) can lead to neuronal cell death and dementia.

Our observation that 4R G55R tau nucleates MT assembly more effectively than does 4R WT tau is completely consistent with the notion that subtle mis-regulation of tau activity can be deleterious. Although it was originally believed that axonal MTs are nucleated at the centrosome in the cell body and then transported as small MTs down the axon [79], more recent work indicates that adult axonal MTs are nucleated acentrosomally [80]. Over-active tau mediated MT nucleation could easily have deleterious effects upon mature neurons. As just one example, aberrant MT nucleation activity could lead to misdirected MT organization, which could in turn interfere with the efficiency of axonal transport. Additionally, although it is unusual for a mutation to increase the effectiveness of a normal activity, the Q336R tau mutation also enhances the ability of tau to promote MT assembly [81]. Further, isoform specific effects of tau mutations such as we observed for MT nucleation are unusual but not unprecedented [52], [75].

Another interesting and non-mutually exclusive possible mechanism of G55R pathogenesis involves release of potentially toxic tau fragments into the extracellular space, which has been observed in dementia patients as well as model systems [82], [83]. This process can be inhibited by the presence of exon 2 [84]. It is possible that the G55R mutation disrupts this inhibitory effect upon secretion of tau fragments, thereby contributing to their accumulation and the pathological process.

It is also interesting that a mutation such as G55R, mapping far from the MT binding region of tau, could have an impact on the ability of the protein to regulate MT nucleation. However, it has long been known that the presence of the amino region of tau can have strong albeit indirect effects upon the ability of the protein to bind to MTs and regulate their dynamics [18], [29], [30]. Additionally, antibody epitope mapping data [85], FRET data [86] and more recent NMR studies [87] indicate that the amino region of tau can fold together with the MT binding region of the protein. It follows that a mutation in the amino region of the protein could have a marked impact on overall three dimensional structure and the ability of tau to engage in intra-molecular and inter-molecular interactions necessary for its action.

## Supporting Information

Figure S1 **The purity of representative recombinant tau preparations is shown by SDS-PAGE and Coomassie staining.** Each panel was taken from a separate gel and corresponds to the final product of an individual tau preparation (see Materials and Methods). In all cases, the migration of the major band in the gel was appropriate for the tau isoform of interest relative to molecular weight standards.
(TIF)

Figure S2 **The G55R mutation does not affect the ability of either 4R tau or 3R tau to influence kinesin mediated microtubule gliding.** Microtubules were assembled with varying ratios of either 3R tau or 4R tau (WT or G55R) to tubulin (containing a small fraction of fluorescent tubulin) and added to kinesin coated cover slips, and kinesin mediated microtubule gliding assayed by fluorescence microscopy. Graphs show mean ± SEM. None of the WT to G55R comparisons were statistically significant by two-tailed t-tests.
(TIF)

Figure S3 **The G55R mutation does not affect the ability of either 4R tau or 3R tau to aggregate.** Quantitative TEM analysis of arachidonic acid induced aggregation reactions were performed for 4R tau and 3R tau in both WT and G55R contexts. Representative images are shown on the left and total aggregate area per field is shown on the right, where bars indicate the mean ± SEM of three independent experiments. No significant difference was found between each G55R mutant and its corresponding WT protein by a two-tailed, unpaired t-test. Scale bar = 500 nm.
(TIF)

Table S1 **The G55R mutation does not affect the ability of either 4R tau or 3R tau to regulate microtubule dynamics.** Plus end microtubule dynamics were recorded for microtubules growing off of axoneme tips and dynamics parameters quantitated as described in the Materials and Methods. No differences between mutant and WT tau proteins were statistically significant by two-tailed t-tests.
(TIF)

## References

[1] Cohn-Hokke PE, Elting MW, Pijnenburg YA, van Swieten JC (2012) Genetics of dementia: update and guidelines for the clinician. Am J Med Genet B Neuropsychiatr Genet 159B: 628–643. 22815225 10.1002/ajmg.b.32080

[2] Seelaar H, Rohrer JD, Pijnenburg YA, Fox NC, van Swieten JC (2011) Clinical, genetic and pathological heterogeneity of frontotemporal dementia: a review. J Neurol Neurosurg Psychiatry 82: 476–486. 20971753 10.1136/jnnp.2010.212225

[3] Rohrer JD, Lashley T, Schott JM, Warren JE, Mead S, et al (2011) Clinical and neuroanatomical signatures of tissue pathology in frontotemporal lobar degeneration. Brain 134: 2565–2581. 21908872 10.1093/brain/awr198 PMC3170537

[4] Rohrer JD, Warren JD (2011) Phenotypic signatures of genetic frontotemporal dementia. Curr Opin Neurol 24: 542–549. 21986680 10.1097/WCO.0b013e32834cd442

[5] Ferrari R, Hardy J, Momeni P (2011) Frontotemporal dementia: from Mendelian genetics towards genome wide association studies. J Mol Neurosci 45: 500–515. 21898125 10.1007/s12031-011-9635-y

[6] Neary D, Snowden JS, Gustafson L, Passant U, Stuss D, et al (1998) Frontotemporal lobar degeneration: a consensus on clinical diagnostic criteria. Neurology 51: 1546–1554. 9855500 10.1212/wnl.51.6.1546

[7] Whitwell JL, Przybelski SA, Weigand SD, Ivnik RJ, Vemuri P, et al (2009) Distinct anatomical subtypes of the behavioural variant of frontotemporal dementia: a cluster analysis study. Brain 132: 2932–2946. 19762452 10.1093/brain/awp232 PMC2768663

[8] Goedert M, Ghetti B, Spillantini MG (2012) Frontotemporal dementia: implications for understanding Alzheimer disease. Cold Spring Harb Perspect Med 2: a006254. 22355793 10.1101/cshperspect.a006254 PMC3281593

[9] Rademakers R, Neumann M, Mackenzie IR (2012) Advances in understanding the molecular basis of frontotemporal dementia. Nat Rev Neurol 8: 423–434. 22732773 10.1038/nrneurol.2012.117 PMC3629543

[10] Clark LN, Poorkaj P, Wszolek Z, Geschwind DH, Nasreddine ZS, et al (1998) Pathogenic implications of mutations in the tau gene in pallido-ponto-nigral degeneration and related neurodegenerative disorders linked to chromosome 17. Proc Natl Acad Sci U S A 95: 13103–13107. 9789048 10.1073/pnas.95.22.13103 PMC23724

[11] Hutton M, Lendon CL, Rizzu P, Baker M, Froelich S, et al (1998) Association of missense and 5′-splice-site mutations in tau with the inherited dementia FTDP-17. Nature 393: 702–705. 9641683 10.1038/31508

[12] Spillantini MG, Murrell JR, Goedert M, Farlow MR, Klug A, et al (1998) Mutation in the tau gene in familial multiple system tauopathy with presenile dementia. Proc Natl Acad Sci U S A 95: 7737–7741. 9636220 10.1073/pnas.95.13.7737 PMC22742

[13] Galimberti D, Scarpini E (2012) Genetics of Frontotemporal Lobar Degeneration. Frontiers in Neurology 3: 1–7. 22536193 10.3389/fneur.2012.00052 PMC3332226

[14] Caceres A, Potrebic S, Kosik KS (1991) The effect of tau antisense oligonucleotides on neurite formation of cultured cerebellar macroneurons. J Neurosci 11: 1515–1523. 1904479 10.1523/JNEUROSCI.11-06-01515.1991 PMC6575418

[15] Esmaeli-Azad B, McCarty JH, Feinstein SC (1994) Sense and antisense transfection analysis of tau function: tau influences net microtubule assembly, neurite outgrowth and neuritic stability. J Cell Sci 107 (Pt 4): 869–879. 10.1242/jcs.107.4.869 8056843

[16] Mandelkow EM, Mandelkow E (2012) Biochemistry and cell biology of tau protein in neurofibrillary degeneration. Cold Spring Harb Perspect Med 2: a006247. 22762014 10.1101/cshperspect.a006247 PMC3385935

[17] Drechsel DN, Hyman AA, Cobb MH, Kirschner MW (1992) Modulation of the dynamic instability of tubulin assembly by the microtubule-associated protein tau. Mol Biol Cell 3: 1141–1154. 1421571 10.1091/mbc.3.10.1141 PMC275678

[18] Trinczek B, Biernat J, Baumann K, Mandelkow EM, Mandelkow E (1995) Domains of tau protein, differential phosphorylation, and dynamic instability of microtubules. Mol Biol Cell 6: 1887–1902. 8590813 10.1091/mbc.6.12.1887 PMC366657

[19] Panda D, Samuel JC, Massie M, Feinstein SC, Wilson L (2003) Differential regulation of microtubule dynamics by three- and four-repeat tau: implications for the onset of neurodegenerative disease. Proc Natl Acad Sci U S A 100: 9548–9553. 12886013 10.1073/pnas.1633508100 PMC170955

[20] Bunker JM, Wilson L, Jordan MA, Feinstein SC (2004) Modulation of microtubule dynamics by tau in living cells: implications for development and neurodegeneration. Mol Biol Cell 15: 2720–2728. 15020716 10.1091/mbc.E04-01-0062 PMC420096

[21] Levy SF, Leboeuf AC, Massie MR, Jordan MA, Wilson L, et al (2005) Three- and four-repeat tau regulate the dynamic instability of two distinct microtubule subpopulations in qualitatively different manners. Implications for neurodegeneration. J Biol Chem 280: 13520–13528. 15671021 10.1074/jbc.M413490200

[22] Conde C, Caceres A (2009) Microtubule assembly, organization and dynamics in axons and dendrites. Nat Rev Neurosci 10: 319–332. 19377501 10.1038/nrn2631

[23] Duncan JE, Goldstein LS (2006) The genetics of axonal transport and axonal transport disorders. PLoS Genet 2: 1275–1284. 10.1371/journal.pgen.0020124 PMC1584265 17009871

[24] Lee G, Thangavel R, Sharma VM, Litersky JM, Bhaskar K, et al (2004) Phosphorylation of tau by fyn: implications for Alzheimer's disease. J Neurosci 24: 2304–2312. 14999081 10.1523/JNEUROSCI.4162-03.2004 PMC6730442

[25] Ittner LM, Ke YD, Delerue F, Bi M, Gladbach A, et al (2010) Dendritic function of tau mediates amyloid-beta toxicity in Alzheimer's disease mouse models. Cell 142: 387–397. 20655099 10.1016/j.cell.2010.06.036

[26] Roberson ED, Halabisky B, Yoo JW, Yao J, Chin J, et al (2011) Amyloid-beta/Fyn-induced synaptic, network, and cognitive impairments depend on tau levels in multiple mouse models of Alzheimer's disease. J Neurosci 31: 700–711. 21228179 10.1523/JNEUROSCI.4152-10.2011 PMC3325794

[27] Lee G, Cowan N, Kirschner M (1988) The primary structure and heterogeneity of tau protein from mouse brain. Science 239: 285–288. 3122323 10.1126/science.3122323

[28] Lee G, Neve RL, Kosik KS (1989) The microtubule binding domain of tau protein. Neuron 2: 1615–1624. 2516729 10.1016/0896-6273(89)90050-0

[29] Butner KA, Kirschner MW (1991) Tau protein binds to microtubules through a flexible array of distributed weak sites. J Cell Biol 115: 717–730. 1918161 10.1083/jcb.115.3.717 PMC2289193

[30] Goode BL, Feinstein SC (1994) Identification of a novel microtubule binding and assembly domain in the developmentally regulated inter-repeat region of tau. J Cell Biol 124: 769–782. 8120098 10.1083/jcb.124.5.769 PMC2119949

[31] Goode BL, Chau M, Denis PE, Feinstein SC (2000) Structural and functional differences between 3-repeat and 4-repeat tau isoforms. Implications for normal tau function and the onset of neurodegenetative disease. J Biol Chem 275: 38182–38189. 10984497 10.1074/jbc.M007489200

[32] Himmler A (1989) Structure of the bovine tau gene: alternatively spliced transcripts generate a protein family. Mol Cell Biol 9: 1389–1396. 2498650 10.1128/mcb.9.4.1389-1396.1989 PMC362555

[33] Gamblin TC, King ME, Dawson H, Vitek MP, Kuret J, et al (2000) In vitro polymerization of tau protein monitored by laser light scattering: method and application to the study of FTDP-17 mutants. Biochemistry 39: 6136–6144. 10821687 10.1021/bi000201f

[34] Barghorn S, Zheng-Fischhofer Q, Ackmann M, Biernat J, von Bergen M, et al (2000) Structure, microtubule interactions, and paired helical filament aggregation by tau mutants of frontotemporal dementias. Biochemistry 39: 11714–11721. 10995239 10.1021/bi000850r

[35] Combs B, Gamblin TC (2012) FTDP-17 Tau Mutations Induce Distinct Effects on Aggregation and Microtubule Interactions. Biochemistry 51: 8597–8607. 23043292 10.1021/bi3010818 PMC3548947

[36] Wolfe MS (2009) Tau mutations in neurodegenerative diseases. J Biol Chem 284: 6021–6025. 18948254 10.1074/jbc.R800013200

[37] Chen J, Kanai Y, Cowan NJ, Hirokawa N (1992) Projection domains of MAP2 and tau determine spacings between microtubules in dendrites and axons. Nature 360: 674–677. 1465130 10.1038/360674a0

[38] King ME, Gamblin TC, Kuret J, Binder LI (2000) Differential assembly of human tau isoforms in the presence of arachidonic acid. J Neurochem 74: 1749–1757. 10737634 10.1046/j.1471-4159.2000.0741749.x

[39] Zhong Q, Congdon EE, Nagaraja HN, Kuret J (2012) Tau isoform composition influences rate and extent of filament formation. J Biol Chem 287: 20711–20719. 22539343 10.1074/jbc.M112.364067 PMC3370253

[40] Carmel G, Mager EM, Binder LI, Kuret J (1996) The structural basis of monoclonal antibody Alz50's selectivity for Alzheimer's disease pathology. J Biol Chem 271: 32789–32795. 8955115 10.1074/jbc.271.51.32789

[41] Brandt R, Leger J, Lee G (1995) Interaction of tau with the neural plasma membrane mediated by tau's amino-terminal projection domain. J Cell Biol 131: 1327–1340. 8522593 10.1083/jcb.131.5.1327 PMC2120645

[42] Ballatore C, Lee VM, Trojanowski JQ (2007) Tau-mediated neurodegeneration in Alzheimer's disease and related disorders. Nat Rev Neurosci 8: 663–672. 17684513 10.1038/nrn2194

[43] Goedert M, Spillantini MG (2006) A century of Alzheimer's disease. Science 314: 777–781. 17082447 10.1126/science.1132814

[44] Makrides V, Shen TE, Bhatia R, Smith BL, Thimm J, et al (2003) Microtubule-dependent oligomerization of tau. Implications for physiological tau function and tauopathies. J Biol Chem 278: 33298–33304. 12805366 10.1074/jbc.M305207200

[45] Rosenberg KJ, Ross JL, Feinstein HE, Feinstein SC, Israelachvili J (2008) Complementary dimerization of microtubule-associated tau protein: Implications for microtubule bundling and tau-mediated pathogenesis. Proc Natl Acad Sci U S A 105: 7445–7450. 18495933 10.1073/pnas.0802036105 PMC2396711

[46] Himmler A, Drechsel D, Kirschner MW, Martin DW Jr (1989) Tau consists of a set of proteins with repeated C-terminal microtubule-binding domains and variable N-terminal domains. Mol Cell Biol 9: 1381–1388. 2498649 10.1128/mcb.9.4.1381-1388.1989 PMC362554

[47] Goedert M, Jakes R (1990) Expression of separate isoforms of human tau protein: correlation with the tau pattern in brain and effects on tubulin polymerization. Embo J 9: 4225–4230. 2124967 10.1002/j.1460-2075.1990.tb07870.x PMC552204

[48] Dolan PJ, Johnson GV (2010) The role of tau kinases in Alzheimer's disease. Curr Opin Drug Discov Devel 13: 595–603. PMC2941661 20812151

[49] Kwon J, Hutton M, Mandelkow E (2009) Tau Mutations Table.

[50] Hong M, Zhukareva V, Vogelsberg-Ragaglia V, Wszolek Z, Reed L, et al (1998) Mutation-specific functional impairments in distinct tau isoforms of hereditary FTDP-17. Science 282: 1914–1917. 9836646 10.1126/science.282.5395.1914

[51] Bunker JM, Kamath K, Wilson L, Jordan MA, Feinstein SC (2006) FTDP-17 mutations compromise the ability of tau to regulate microtubule dynamics in cells. J Biol Chem 281: 11856–11863. 16495230 10.1074/jbc.M509420200

[52] LeBoeuf AC, Levy SF, Gaylord M, Bhattacharya A, Singh AK, et al.. (2008) FTDP-17 mutations in tau alter the regulation of microtubule dynamics - an “Alternative Core” model for normal and pathological tau action. J Biol Chem Oct 21 [Epub ahead of print]. 10.1074/jbc.M803519200 PMC2606000 18940799

[53] Michaelis ML, Ansar S, Chen Y, Reiff ER, Seyb KI, et al (2005) {beta}-Amyloid-Induced Neurodegeneration and Protection by Structurally Diverse Microtubule-Stabilizing Agents. J Pharmacol Exp Ther 312: 659–668. 15375176 10.1124/jpet.104.074450

[54] Brunden KR, Zhang B, Carroll J, Yao Y, Potuzak JS, et al (2010) Epothilone D improves microtubule density, axonal integrity, and cognition in a transgenic mouse model of tauopathy. J Neurosci 30: 13861–13866. 20943926 10.1523/JNEUROSCI.3059-10.2010 PMC2958430

[55] Zhang B, Carroll J, Trojanowski JQ, Yao Y, Iba M, et al (2012) The microtubule-stabilizing agent, epothilone D, reduces axonal dysfunction, neurotoxicity, cognitive deficits, and Alzheimer-like pathology in an interventional study with aged tau transgenic mice. J Neurosci 32: 3601–3611. 22423084 10.1523/JNEUROSCI.4922-11.2012 PMC3321513

[56] Hayashi S, Toyoshima Y, Hasegawa M, Umeda Y, Wakabayashi K, et al (2002) Late-onset frontotemporal dementia with a novel exon 1 (Arg5His) tau gene mutation. Ann Neurol 51: 525–530. 11921059 10.1002/ana.10163

[57] Poorkaj P, Muma NA, Zhukareva V, Cochran EJ, Shannon KM, et al (2002) An R5L tau mutation in a subject with a progressive supranuclear palsy phenotype. Ann Neurol 52: 511–516. 12325083 10.1002/ana.10340

[58] Rizzu P, Van Swieten JC, Joosse M, Hasegawa M, Stevens M, et al (1999) High prevalence of mutations in the microtubule-associated protein tau in a population study of frontotemporal dementia in the Netherlands. Am J Hum Genet 64: 414–421. 9973279 10.1086/302256 PMC1377751

[59] Goedert M, Ghetti B, Spillantini MG (2000) Tau gene mutations in frontotemporal dementia and parkinsonism linked to chromosome 17 (FTDP-17). Their relevance for understanding the neurogenerative process. Ann N Y Acad Sci 920: 74–83. 11193179 10.1111/j.1749-6632.2000.tb06907.x

[60] Neumann M, Schulz-Schaeffer W, Crowther RA, Smith MJ, Spillantini MG, et al (2001) Pick's disease associated with the novel Tau gene mutation K369I. Ann Neurol 50: 503–513. 11601501 10.1002/ana.1223

[61] Delobel P, Flament S, Hamdane M, Jakes R, Rousseau A, et al (2002) Functional characterization of FTDP-17 tau gene mutations through their effects on Xenopus oocyte maturation. J Biol Chem 277: 9199–9205. 11756436 10.1074/jbc.M107716200

[62] Zekanowski C, Peplonska B, Styczynska M, Gustaw K, Kuznicki J, et al (2003) Mutation screening of the MAPT and STH genes in Polish patients with clinically diagnosed frontotemporal dementia. Dement Geriatr Cogn Disord 16: 126–131. 12826737 10.1159/000070999

[63] Baker M, Litvan I, Houlden H, Adamson J, Dickson D, et al (1999) Association of an extended haplotype in the tau gene with progressive supranuclear palsy. Hum Mol Genet 8: 711–715. 10072441 10.1093/hmg/8.4.711

[64] Pittman AM, Myers AJ, Abou-Sleiman P, Fung HC, Kaleem M, et al (2005) Linkage disequilibrium fine mapping and haplotype association analysis of the tau gene in progressive supranuclear palsy and corticobasal degeneration. J Med Genet 42: 837–846. 15792962 10.1136/jmg.2005.031377 PMC1735957

[65] Kiris E, Ventimiglia D, Sargin ME, Gaylord MR, Altinok A, et al (2011) Combinatorial Tau pseudophosphorylation: markedly different regulatory effects on microtubule assembly and dynamic instability than the sum of the individual parts. J Biol Chem 286: 14257–14270. 21288907 10.1074/jbc.M111.219311 PMC3077627

[66] Peck A, Sargin ME, LaPointe NE, Rose K, Manjunath BS, et al (2011) Tau isoform-specific modulation of kinesin-driven microtubule gliding rates and trajectories as determined with tau-stabilized microtubules. Cytoskeleton (Hoboken) 68: 44–55. 21162159 10.1002/cm.20494

[67] Miller HP, Wilson L (2010) Preparation of Microtubule Protein and Purified Tubulin from Bovine Brain by Cycles of Assembly and Disassembly and Phosphocellulose Chromatography. In: Wilson L, Correia, J.J., Methods in Cell Biology: Elsevier Inc. pp. 3–15. 10.1016/S0091-679X(10)95001-2 20466126

[68] Hyman A, Drechsel D, Kellogg D, Salser S, Sawin K, et al (1991) Preparation of modified tubulins. Methods Enzymol 196: 478–485. 2034137 10.1016/0076-6879(91)96041-o

[69] Yenjerla M, Lopus M, Wilson L (2010) Analysis of dynamic instability of steady-state microtubules in vitro by video-enhanced differential interference contrast microscopy with an appendix by Emin Oroudjev. Methods Cell Biol 95: 189–206. 20466136 10.1016/S0091-679X(10)95011-5

[70] Lopus M, Oroudjev E, Wilson L, Wilhelm S, Widdison W, et al (2010) Maytansine and cellular metabolites of antibody-maytansinoid conjugates strongly suppress microtubule dynamics by binding to microtubules. Mol Cancer Ther 9: 2689–2699. 20937594 10.1158/1535-7163.MCT-10-0644 PMC2954514

[71] Mandelkow E, von Bergen M, Biernat J, Mandelkow EM (2007) Structural principles of tau and the paired helical filaments of Alzheimer's disease. Brain Pathol 17: 83–90. 17493042 10.1111/j.1750-3639.2007.00053.x PMC8095506

[72] Cotton RG, Scriver CR (1998) Proof of “disease causing” mutation. Hum Mutat 12: 1–3. 9633813 10.1002/(SICI)1098-1004(1998)12:1<1::AID-HUMU1>3.0.CO;2-M

[73] Antonarakis SE, Cooper DN (2010) Human Gene Mutation: Mechanisms and Consequences. In: Speicher M, Antonarakis SE, Motulsky AG, Vogel and Motulsky's Human Genetics. 4th ed. Berlin Heidelberg: Springer. pp. 319–364.

[74] Guerreiro RJ, Baquero M, Blesa R, Boada M, Bras JM, et al (2010) Genetic screening of Alzheimer's disease genes in Iberian and African samples yields novel mutations in presenilins and APP. Neurobiol Aging 31: 725–731. 18667258 10.1016/j.neurobiolaging.2008.06.012 PMC2850052

[75] Hogg M, Grujic ZM, Baker M, Demirci S, Guillozet AL, et al (2003) The L266V tau mutation is associated with frontotemporal dementia and Pick-like 3R and 4R tauopathy. Acta Neuropathol (Berl) 106: 323–336. 12883828 10.1007/s00401-003-0734-x

[76] Kertesz A (2010) Frontotemporal dementia, Pick's disease. Ideggyogy Sz 63: 4–12. 20420119

[77] Mi K, Johnson GV (2006) The role of tau phosphorylation in the pathogenesis of Alzheimer's disease. Curr Alzheimer Res 3: 449–463. 17168644 10.2174/156720506779025279

[78] Feinstein SC, Wilson L (2005) Inability of tau to properly regulate neuronal microtubule dynamics: a loss-of-function mechanism by which tau might mediate neuronal cell death. Biochim Biophys Acta 1739: 268–279. 15615645 10.1016/j.bbadis.2004.07.002

[79] Yu W, Centonze VE, Ahmad FJ, Baas PW (1993) Microtubule nucleation and release from the neuronal centrosome. J Cell Biol 122: 349–359. 8320258 10.1083/jcb.122.2.349 PMC2119640

[80] Stiess M, Maghelli N, Kapitein LC, Gomis-Ruth S, Wilsch-Brauninger M, et al (2010) Axon extension occurs independently of centrosomal microtubule nucleation. Science 327: 704–707. 20056854 10.1126/science.1182179

[81] Pickering-Brown SM, Baker M, Nonaka T, Ikeda K, Sharma S, et al (2004) Frontotemporal dementia with Pick-type histology associated with Q336R mutation in the tau gene. Brain 127: 1415–1426. 15047590 10.1093/brain/awh147

[82] Borroni B, Gardoni F, Parnetti L, Magno L, Malinverno M, et al (2009) Pattern of Tau forms in CSF is altered in progressive supranuclear palsy. Neurobiol Aging 30: 34–40. 17709155 10.1016/j.neurobiolaging.2007.05.009

[83] Kim W, Lee S, Jung C, Ahmed A, Lee G, et al (2010) Interneuronal transfer of human tau between Lamprey central neurons in situ. J Alzheimers Dis 19: 647–664. 20110609 10.3233/JAD-2010-1273

[84] Kim W, Lee S, Hall GF (2010) Secretion of human tau fragments resembling CSF-tau in Alzheimer's disease is modulated by the presence of the exon 2 insert. FEBS Lett 584: 3085–3088. 20553717 10.1016/j.febslet.2010.05.042

[85] Jicha GA, Bowser R, Kazam IG, Davies P (1997) Alz-50 and MC-1, a new monoclonal antibody raised to paired helical filaments, recognize conformational epitopes on recombinant tau. J Neurosci Res 48: 128–132. 9130141 10.1002/(sici)1097-4547(19970415)48:2<128::aid-jnr5>3.0.co;2-e

[86] Jeganathan S, von Bergen M, Brutlach H, Steinhoff HJ, Mandelkow E (2006) Global hairpin folding of tau in solution. Biochemistry 45: 2283–2293. 16475817 10.1021/bi0521543

[87] Mukrasch MD, Bibow S, Korukottu J, Jeganathan S, Biernat J, et al (2009) Structural polymorphism of 441-residue tau at single residue resolution. PLoS Biol 7: e34. 19226187 10.1371/journal.pbio.1000034 PMC2642882

