# Supplementary figures and images for: A Novel MAPT Mutation, G55R, in a Frontotemporal Dementia Patient Leads to Altered Tau Function
Source: PLoS One. 2013 Sep 27;8(9):e76409. doi: 10.1371/journal.pone.0076409 (PMC3785453; doi:10.1371/journal.pone.0076409)

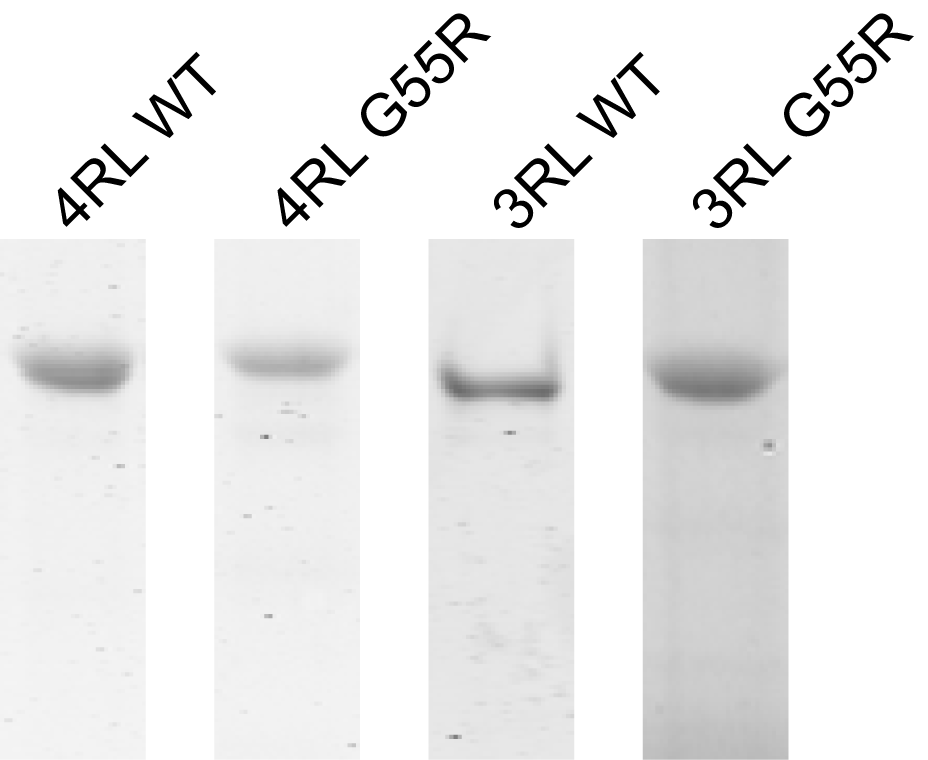

Supplement: Figure S1 — The purity of representative recombinant tau preparations is shown by SDS-PAGE and Coomassie staining. Each panel was taken from a separate gel and corresponds to the final product of an individual tau preparation (see Materials and Methods). In all cases, the migration of the major band in the gel was appropriate for the tau isoform of interest relative to molecular weight standards. (TIF) [file pone.0076409.s001.tif]

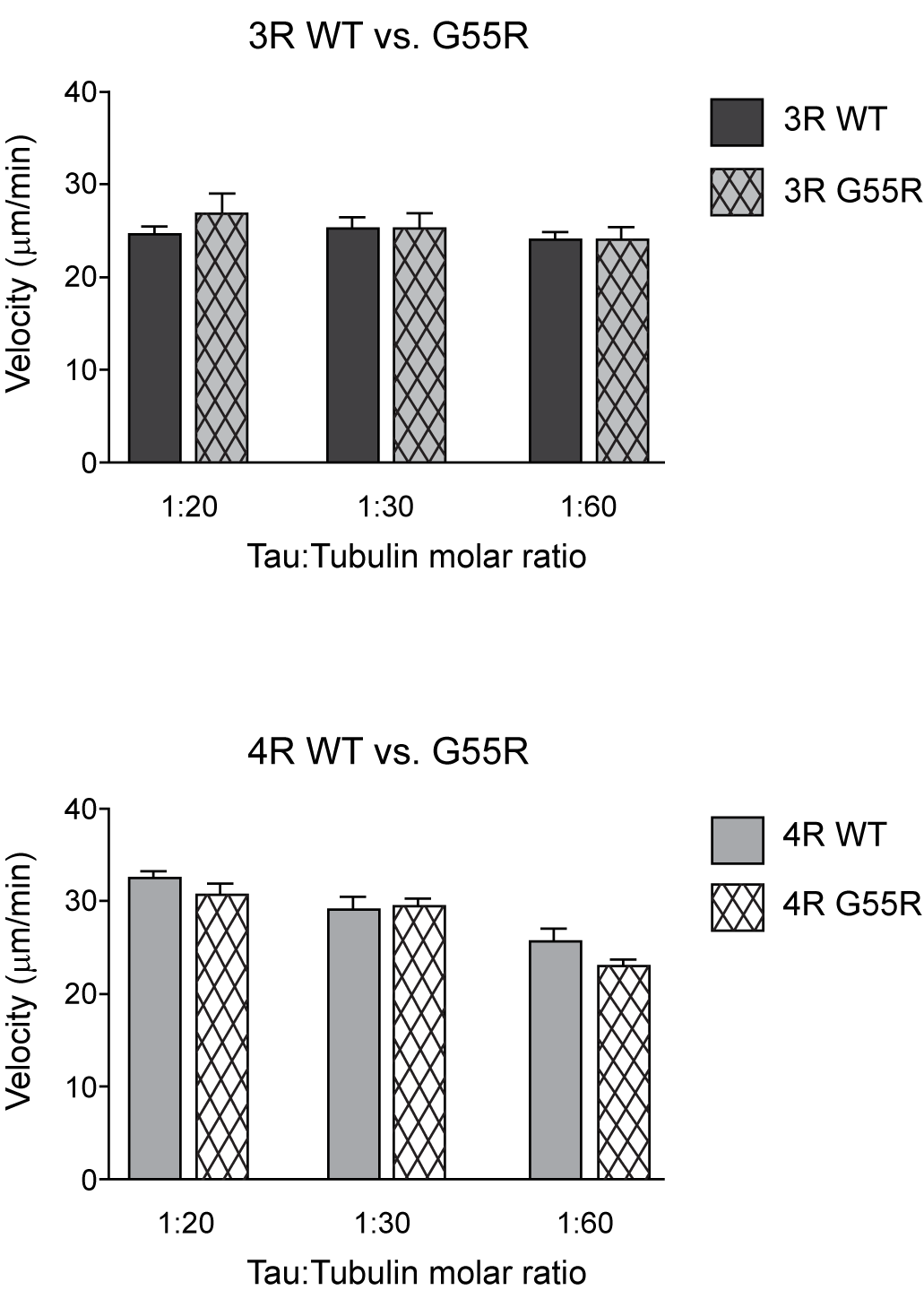

Supplement: Figure S2 — The G55R mutation does not affect the ability of either 4R tau or 3R tau to influence kinesin mediated microtubule gliding. Microtubules were assembled with varying ratios of either 3R tau or 4R tau (WT or G55R) to tubulin (containing a small fraction of fluorescent tubulin) and added to kinesin coated cover slips, and kinesin mediated microtubule gliding assayed by fluorescence microscopy. Graphs show mean ± SEM. None of the WT to G55R comparisons were statistically significant by two-tailed t-tests. (TIF) [file pone.0076409.s002.tif]

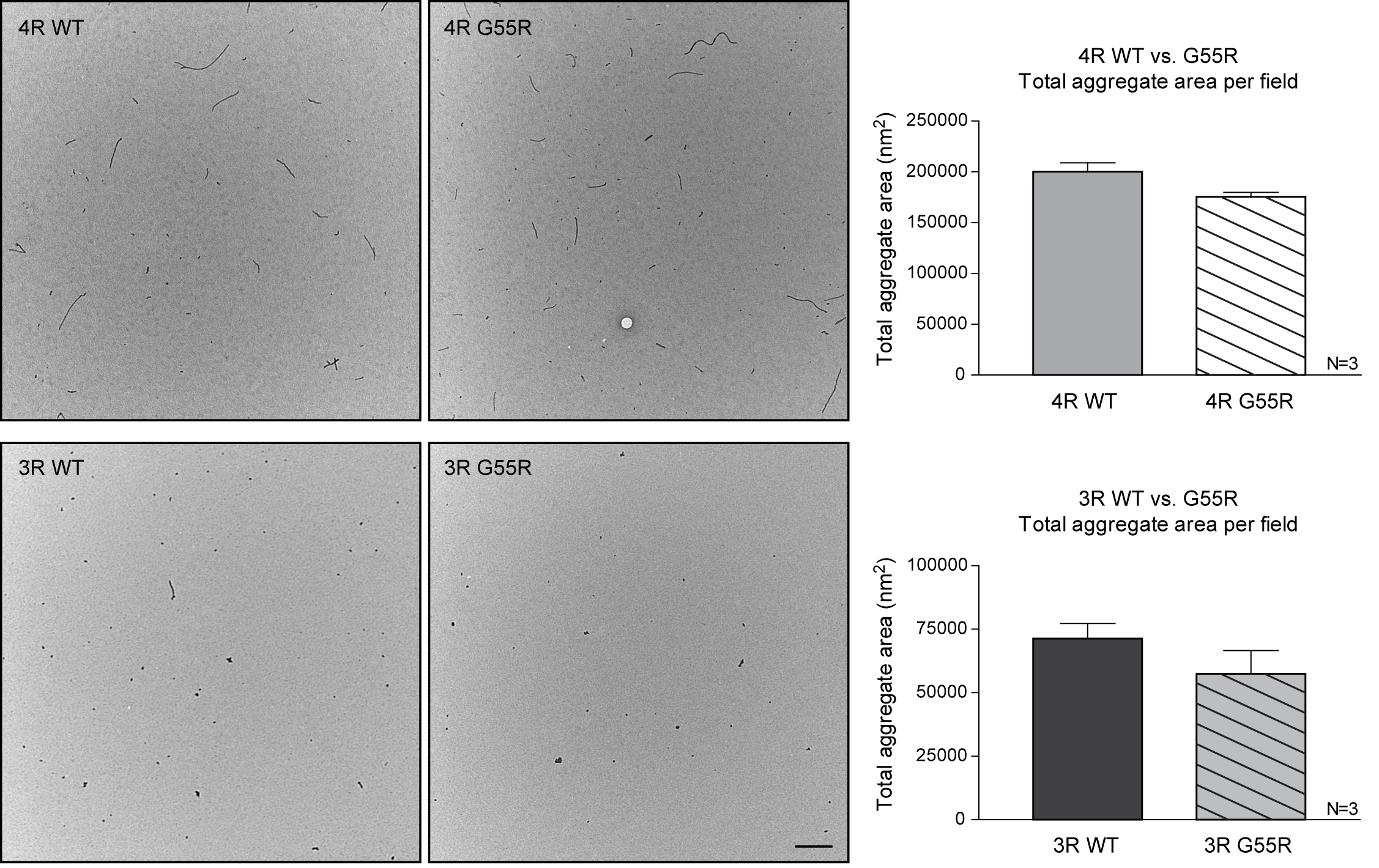

Supplement: Figure S3 — The G55R mutation does not affect the ability of either 4R tau or 3R tau to aggregate. Quantitative TEM analysis of arachidonic acid induced aggregation reactions were performed for 4R tau and 3R tau in both WT and G55R contexts. Representative images are shown on the left and total aggregate area per field is shown on the right, where bars indicate the mean ± SEM of three independent experiments. No significant difference was found between each G55R mutant and its corresponding WT protein by a two-tailed, unpaired t-test. Scale bar = 500 nm. (TIF) [file pone.0076409.s003.tif]

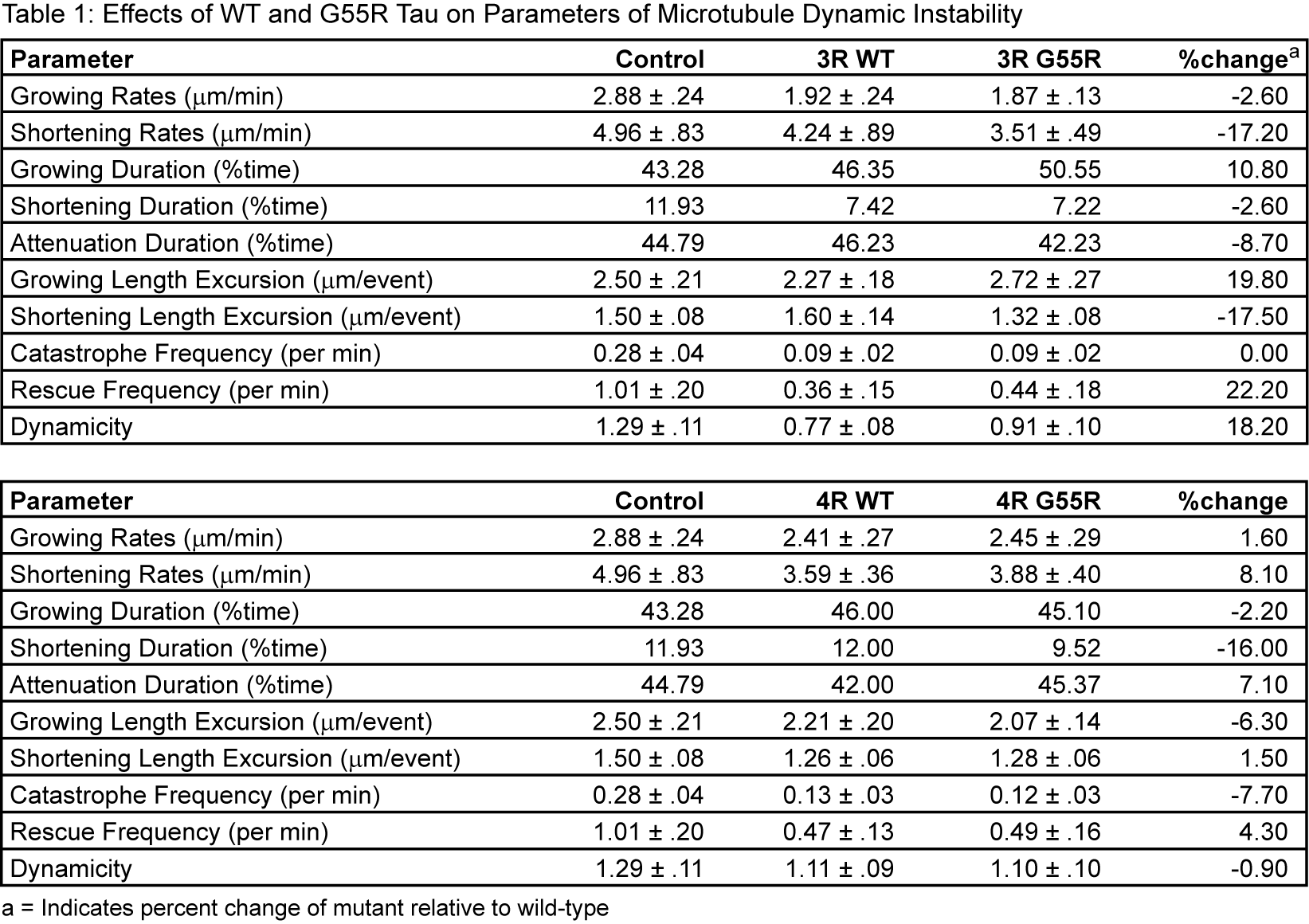

Supplement: Table S1 — The G55R mutation does not affect the ability of either 4R tau or 3R tau to regulate microtubule dynamics. Plus end microtubule dynamics were recorded for microtubules growing off of axoneme tips and dynamics parameters quantitated as described in the Materials and Methods. No differences between mutant and WT tau proteins were statistically significant by two-tailed t-tests. (TIF) [file pone.0076409.s004.tif]
